# OTS964, a TOPK Inhibitor, Is Susceptible to ABCG2-Mediated Drug Resistance

**DOI:** 10.3389/fphar.2021.620874

**Published:** 2021-02-15

**Authors:** Yuqi Yang, Zhuo-Xun Wu, Jing-Quan Wang, Qiu-Xu Teng, Zi-Ning Lei, Sabrina Lusvarghi, Suresh V. Ambudkar, Zhe-Sheng Chen, Dong-Hua Yang

**Affiliations:** ^1^ Department of Pharmaceutical Sciences, College of Pharmacy and Health Sciences, St. John’s University, Queens, NY, United States; ^2^ Laboratory of Cell Biology, Center for Cancer Research, National Cancer Institute, NIH, Bethesda, MD, United States

**Keywords:** TOPK inhibitor, OTS964, ABCG2, ABC transporter, cancer

## Abstract

OTS964 is a potent T-LAK cell-originated protein kinase (TOPK) inhibitor. Herein, we investigated the interaction of OTS964 and multidrug resistance (MDR)-associated ATP-binding cassette sub-family G member 2 (ABCG2). The cell viability assay indicated that the effect of OTS964 is limited in cancer drug-resistant and transfected cells overexpressing ABCG2. We found that the known ABCG2 transporter inhibitor has the ability to sensitize ABCG2-overexpressing cells to OTS964. In mechanism-based studies, OTS964 shows inhibitory effect on the efflux function mediated by ABCG2, and in turn, affects the pharmacokinetic profile of other ABCG2 substrate-drugs. Furthermore, OTS964 upregulates ABCG2 protein expression, resulting in enhanced resistance to ABCG2 substrate-drugs. The ATPase assay demonstrated that OTS964 stimulates ATPase activity of ABCG2 in a concentration-dependent manner. The computational molecular docking analysis combined with results from ATPase assay suggested that OTS964 interacts with drug-binding pocket of ABCG2 and has substrate-like behaviors. Thus, OTS964 is an MDR-susceptible agent due to its interactions with ABCG2, and overexpression of ABCG2 transporter may attenuate its therapeutic effect in cancer cells.

## Introduction

T-LAK cell-originated protein kinase (TOPK) is a mitogen-activated protein kinase-like kinase (MAPKK), which plays a critical role in facilitating cell cycle control and mitotic progression (Herbert et al., 2018). TOPK expression is largely confined to tissues with rapid cell proliferation, and TOPK mRNA can be abundantly detected in tissues derived from testis, placenta, brain, and thymus (Herbert et al., 2018). Dysregulated expression of TOPK often results in cancer development and tumor metastasis in various cancer types (Hansel et al., 2009; Shih et al., 2012; Joel et al., 2015; Ikeda et al., 2016; Jiang et al., 2019). TOPK overexpression promotes cell growth and induces tumor formation (Zhu et al., 2007). Conversely, downregulation of TOPK expression suppresses tumor growth, migration, and invasion (Gao et al., 2019). Therefore, TOPK may be a potential cancer-specific biomarker, and serves as a druggable cancer target with minimal harm to normal tissue (Bellayr et al., 2016; Hayashi et al., 2018; Herbert et al., 2018; Pirovano et al., 2020).

**FIGURE 1:**
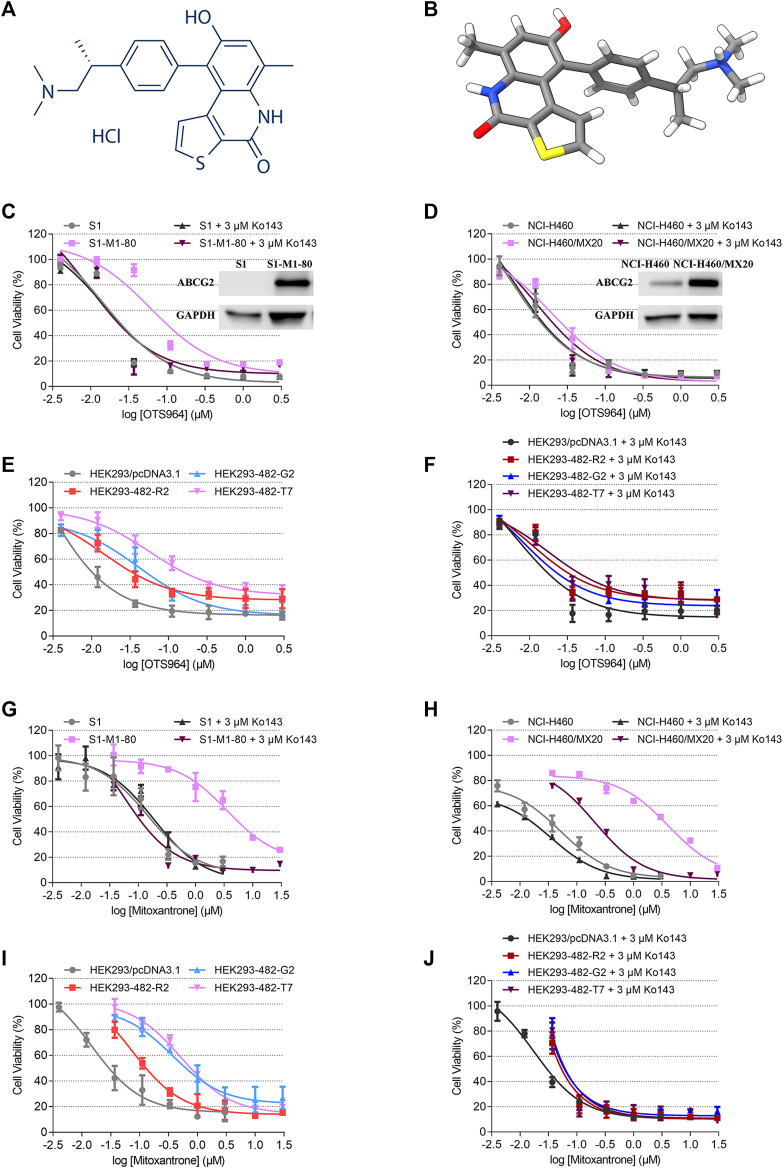
Chemical structure of OTS964, and the cell viability-concentration curves for OTS964 and mitoxantrone in MDR cells mediated by ABCG2 and their counterparts in parental cells. **(A)** 2D view of OTS964 structure. **(B)** 3D view of OTS964 structure. OTS964 molecule is exhibited as colored sticks. Gray: carbon; white: hydrogen; red: oxygen; blue: nitrogen; yellow: sulfur. The cytotoxic activity of OTS964 in **(C)** S1-M1-80 and S1, **(D)** NCI-H460/MX20 and NCI-H460, and **(E, F)** ABCG2-transfected HEK293 cells (HEK293/ABCG2-482-R2, HEK293/ABCG2-482-G2, and HEK293/ABCG2-482-T7) and HEK293/pcDNA3.1 co-treated without/with Ko143. The cytotoxic activity of mitoxantrone in **(G)** S1-M1-80 and S1, **(H)** NCI-H460/MX20 and NCI-H460, and **(I, J)** ABCG2-transfected HEK293 cells (HEK293/ABCG2-482-R2, HEK293/ABCG2-482-G2, and HEK293/ABCG2-482-T7) and HEK293/pcDNA3.1 co-treated without/with Ko143. Ko143 served as a known ABCG2 inhibitor. Each dot is displayed as mean ± SD obtained from three experiments performed independently.

A small molecule TOPK inhibitor, OTS964, has been reported to have the ability to suppress tumor growth and to induce apoptotic cell death in various cancer models both *in vitro* and *in vivo* (Matsuo et al., 2014; Ikeda et al., 2016; Pirovano et al., 2017). The chemical structure of OTS964 is presented in Figures 1A,B. Most recently, Pirovano et al. developed a [^18^F]-labeled OTS964 and showed that it has acceptable pharmacokinetic profile with favorable biodistribution in a mouse model (Pirovano et al., 2019). Free form of OTS964 has unfavorable hematopoietic toxicity; however, encapsulated OTS964 liposomes could overcome this problem (Gilabert-Oriol et al., 2019). This is a promising step toward clinical use of OTS964.

Evidence from clinical contexts have indicated that the efficacy of anticancer drugs is restricted by multidrug resistance (MDR) (Holohan et al., 2013). Pharmacodynamic and/or pharmacokinetic resistance can further confer limited effectiveness of cytotoxic and targeted drugs (Vagiannis et al., 2020). Dysregulation or mutation of therapeutic target results in pharmacodynamic resistance; while, enhanced efflux function or drug deactivation via an alternative metabolic pathway promotes pharmacokinetic resistance (Holohan et al., 2013). ATP-binding cassette (ABC) transporters, distributed in the lipid raft of certain cells, mediate drug efflux to attenuate intracellular level of chemotherapeutic drugs from accumulating in cancer cells (Klappe et al., 2009). ABC sub-family G member 2 (ABCG2, breast cancer resistance protein/BCRP) is a common factor responsible for MDR (Theodoulou and Kerr, 2015). It is known that ABCG2 is expressed on the breast, ovaries, testis, placenta, intestine, liver, and blood brain barrier (Szakács et al., 2008; Manolaridis et al., 2018).

TOPK and ABCG2 share similar tissue distributions. Hence, we investigated whether ABCG2 could restrict the effectiveness of TOPK inhibitors. In particular, we assessed the antitumor efficacy of OTS964 in the presence of ABCG2 in cancer cells.

## Materials and Methods

### Chemicals and Reagents

OTS964 was kindly provided as a gift by ChemieTek company (Indianapolis, IN). The chemical purity of OTS964 is >99.5% (HPLC at 214 and 254 nm). Fetal bovine serum was obtained from Atlanta Biologicals (Minneapolis, MN). Dulbecco’s modified Eagle medium, antibiotics (penicillin/streptomycin), and trypsin-EDTA were purchased from Corning (Corning, NY). Topotecan was obtained from Selleckchem (Houston, TX). Ko143, G418, and cisplatin were purchased from Enzo Life Sciences (Farmingdale, NY). SN-38, and mitoxantrone were obtained from Medkoo Biosciences (Morrisville, NC). DMSO, MTT, and anti-BCRP antibody (BXP-21) were purchased from Millipore-Sigma (Burlington, MA). HRP-conjugated secondary antibody was obtained from Cell Signaling Technology (Dancers, MA). [^3^H]-Mitoxantrone (11 μCi/mmol) was obtained from Moravek Biochemicals (Brea, CA). Anti-GAPDH antibody (GA1R), liquid scintillation cocktail, and all other reagents were obtained from Thermo Fisher Scientific (Waltham, MA).

### Cell Lines and Cell Culture

Mitoxantrone-selected MDR cell lines expressing ABCG2, NCI-H460/MX20 and S1-M1-80, were developed in medium with mitoxantrone at 20 nM and 80 μM concentrations, respectively. Their respective parental cell lines are non-small cell lung cancer cell line NCI-H460 and human colon carcinoma cell line S1. NCI-H460/MX20 cells were shown to overexpress wild-type ABCG2 protein (Robey et al., 2001; Henrich et al., 2006), while S1-M1-80 cells were shown to overexpress a mutant allele (R482G) in *ABCG2* gene (Miyake et al., 1999; Honjo et al., 2001). HEK293/pcDNA3.1, HEK293/ABCG2-482-R2, HEK293/ABCG2-482-G2, and HEK293/ABCG2-482-T7 were transfected with either an empty vector pcDNA3.1 or a pcDNA3.1 vector containing a full length ABCG2 encoding arginine (R), glycine (G), or threonine (T) at position 482 (Robey et al., 2003). All transfected cell lines were selected with medium containing 2 mg/ml G418. All cells were cultured in complete medium at 37°C in a humidified incubator supplied with 5% CO_2_. All MDR cells were cultured in drug-free complete medium for at least 3 weeks and passaged for at least three generations before further experimental use.

### Cell Viability Assay

As previously described (Jiang et al., 2019), an MTT assay was used to examine cell viability rate after treatment with OTS964 and other chemotherapeutic drugs. Briefly, 5 × 10^3^–7 × 10^3^ cells/well were evenly seeded into a 96-well plate. The next day, serial concentrations of substrate-drugs were added to designated wells with or without 2 h pretreatment of OTS964 or known ABCG2 inhibitor at indicated concentrations. After a 72 h incubation period, an MTT solution was added following 3 h incubation at 37°C in the dark. The supernatant was discarded, and this was followed by the addition of DMSO to dissolve resulting formazan crystals. The absorbance was measured at 570 nm using an UV/Vis microplate spectrophotometer (Fisher Sci. Fair Lawn, NJ). The log scale curves in GraphPad (log inhibitor vs responses) were used to fit cell viability curves. Resistance fold (RF) was calculated by dividing the IC_50_ values for antineoplastic drugs of drug-sensitive cells without inhibitor by the IC_50_ values for chemotherapeutic drugs of drug-sensitive cells with inhibitor or drug-resistant cells with or without inhibitor.

### Western Blot Analysis

A Western blot was conducted to determine protein expression level by using an established protocol (Ji et al., 2018b). Briefly, following incubation with OTS964 at indicated concentrations for a serial time-course, lysates were collected and quantified by a BCA protein kit. Equal amounts of total protein (10–20 μg) were loaded and separated by SDS-PAGE, followed by transfer onto a PVDF membrane. After blocking with 5% non-fat milk for 2 h at room temperature, membrane was incubated overnight with primary antibodies at 4°C. The next day, after washing with TBST, the membrane was incubated with an HRP-conjugated secondary antibody for 2 h at room temperature. Subsequently, the chemiluminescence signal of protein-antibody complex was visualized by ECL substrate as per manufacturer’s instructions. The relative density of each protein band was analyzed by Fiji software for Windows (NIH, Bethesda, MD).

### Accumulation Assay

Tritium-labeled mitoxantrone accumulation assay was performed to assess the transport function mediated by MDR-associated ABC transporters (Ji et al., 2018a). Each cell line was seeded evenly into a 24-well plate with a density of 1 × 10^6^ cells/well. The next day, cells were pretreated for 2 h with or without OTS964 or positive ABCG2 inhibitor at indicated concentrations. Thereafter, tritium-labeled substrate-drug was added to designated wells and incubated for 2 h. Followed by washing with ice-cold PBS, cells were harvested and transferred into scintillation fluid. A Packard TRI-CARB 1900 CA liquid scintillation analyzer (Packard Instrument, Downers Grove, IL) was used to measure radioactivity.

### ATPase Assay

The vanadate-sensitive ATPase activity of ABCG2 was measured, as previously described, based on the amount of inorganic phosphate (P_i_) produced from hydrolyzed ATP (Yang et al., 2020). The amount of P_i_ was quantified by a modified colorimetric method of Murphy and Riley (Ojida et al., 2004).

### Molecular Docking of OTS964 With Human ABCG2 Model

The OTS964 3D structure was constructed for docking simulation as previously described (Wang et al., 2020). Human ABCG2 6VXI (mitoxantrone-bound) (Orlando and Liao, 2020) were obtained from RCSB Protein Data Bank (PDB). The protein model is inward facing. Docking calculations were performed in AutoDock Vina (version 1.1.2) (Trott and Olson, 2010). Hydrogen atoms and partial charges were added using AutoDockTools (ADT, version 1.5.4). Docking grid center coordinates were determined from the bound ligands provided in PDB files. Receptor/ligand preparation and docking simulation were performed using default settings. The top-scoring pose (sorted by affinity score: kcal/mol) was selected for further analysis and visualization.

### Statistics

All data are presented as mean ± SD. The *p* values were computed by one-way or two-way ANOVA following Tukey post hoc analysis, if appropriate. Data analysis and follow-up statistical evaluation were performed by GraphPad software for Windows (San Diego, CA). The *a priori* significance level was *p* < 0.05.

## Results

### Antineoplastic efficacy of OTS964 was compromised by the presence of ABCG2.

An MTT assay was performed to examine the susceptibility of OTS964 to MDR mediated by ABCG2. Herein, RF value was used to evaluate the degree of increased resistance to OTS964 resulting from the presence of ABCG2 transporter. Based on Figures 1C,D the drug sensitivity of OTS964 was attenuated in S1-M1-80 and NCI-H460/MX20 cell lines by 4.16- and 3.64-fold, respectively, relative to their corresponding parental cell lines. Also, cells transfected with wild-type (R482) or mutant (R482T or R482G) ABCG2 were used. This is because Robey et al. showed that variations at amino-acid 482 in the *ABCG2* gene affect the substrate specificity of the protein, for example rhodamine 123 and daunorubicin are transported only by mutant ABCG2; also, mutant ABCG2 confers higher level of resistance to certain substrate-drug such as mitoxantrone compared to wild type (Robey et al., 2003). Our results showed that the effectiveness of OTS964 was limited by 6.39-, 18.41-, and 25.41-fold, respectively, in HEK293/ABCG2-482-R2, HEK293/ABCG2-482-G2, and HEK293/ABCG2-482-T7 cells, compared with counterparts in HEK293/pcDNA3.1 cells (Figure 1E). Notably, Ko143, a known ABCG2 inhibitor, had the ability to restore sensitivity of OTS964 in ABCG2-overexpressing cell lines (Figures 1C–F). Meanwhile, mitoxantrone served as a reference ABCG2 substrate. The RF values for S1-M1-80, NCI-H460/MX20, HEK293/ABCG2-482-R2, HEK293/ABCG2-482-G2, and HEK293/ABCG2-482-T7 cell lines were 19.57, 79.40, 5.03, 24.49 and 38.90, respectively (Figures 1G–I). Antineoplastic activity of mitoxantrone was sensitized by Ko143 in MDR cell lines mediated by ABCG2, as shown in Figures 1G–J. Considering different responses in wild-type (R482) and mutant (R482G and R482T) ABCG2, gene-transfected HEK293 cell lines were used for further studies. These results implicate that ABCG2 transporter could confer resistance to OTS964.

### OTS964 did not antagonize MDR mediated by ABCG2.

Some repurposed compounds behave as chemosensitizers or substrates based on different cellular settings caused by ABC transporters (Brózik et al., 2011; Beretta et al., 2017). Hence, an MTT assay was performed to examine the ability of OTS964 to restore drug sensitivity in cell lines with MDR mediated by ABCG2. To prevent overlapping impact due to the high cytotoxic nature of OTS964 in drug-sensitive cell lines, this study was conducted only on drug-resistant cell lines. According to the cell viability curves in Figures 1C–E, the maximum non-toxic concentration (the concentration at which cell viability rate was more than 80%) was 10 nM in MDR cell lines. In this section, the value of RF was used to assess the ability of OTS964 or known ABCG2 inhibitor to antagonize MDR mediated by ABCG2. Table 1 summarized the IC_50_ and RF values of anticancer drugs with or without OTS964 or a known ABCG2 inhibitor Ko143 at non-toxic concentrations. Interestingly, in ABCG2-mediated MDR cells, OTS964 induced resistance to mitoxantrone in S1-M1-80 cell line, as evidenced by increased RF value for mitoxantrone from 19.57-fold to 57.63-fold relative to S1-M1-80 cells without an inhibitor. However, OTS964 at 10 nM did not significantly affect IC_50_ values for topotecan or SN-38 in S1-M1-80 cells relative to counterparts in S1-M1-80 cells without an inhibitor. Also, OTS964 showed an effect similar to mitoxantrone and SN-38 in ABCG2-transfected cell lines, but not to topotecan. In contrast, Ko143 as a positive ABCG2 inhibitor reversed substrate resistance. Notably, OTS964 and positive inhibitor did not affect cell viability of non-substrate drug cisplatin. These results demonstrated that OTS964 cannot restore drug efficacy in MDR cell lines, but may induce MDR mediated by ABCG2.

**TABLE 1:** The effect of OTS964 on the anticancer efficacy of chemotherapeutic drugs in drug-selected and gene-transfected ABCG2-overexpressing cell lines.

Treatment	IC_50_^a^ ± SD (μM) (RF^b^)					
	S1	S1-M1-80	HEK293/pcDNA3.1	HEK293/ABCG2-482-R2	HEK293/ABCG2-482-G2	HEK293/ABCG2-482-T7
Mitoxantrone	0.19 ± 0.13 (1.00)	3.75 ± 0.78 (19.57)*	0.02 ± 0.01 (1.00)	0.08 ± 0.02 (5.03)*	0.37 ± 0.03 (24.49)*	0.59 ± 0.14 (38.90)*
+OTS964 5 nM	-	11.79 ± 0.08 (61.47)*	-	0.09 ± 0.03 (6.10)*	0.51 ± 0.15 (34.11)*	0.77 ± 0.21 (50.81)*
+OTS964 10 nM	-	11.05 ± 0.41 (57.63)*	-	0.14 ± 0.01 (9.07)*	0.66 ± 0.09 (43.77)*	0.80 ± 0.13 (53.29)*
+Ko143 3 μM	0.20 ± 0.07 (1.05)	0.17 ± 0.10 (0.91)	0.02 ± 0.01 (1.20)	0.04 ± 0.01 (2.68)	0.06 ± 0.03 (3.72)	0.07 ± 0.01 (4.48)
Topotecan	0.04 ± 0.01 (1.00)	0.87 ± 0.29 (19.72)*	0.03 ± 0.02 (1.00)	0.19 ± 0.06 (6.05)*	1.21 ± 0.05 (38.44)*	1.85 ± 0.69 (58.74)*
+OTS964 5 nM	-	0.94 ± 0.28 (21.51)*	-	0.17 ± 0.04 (5.25)*	1.35 ± 0.07 (42.69)*	1.85 ± 0.49 (58.67)*
+OTS964 10 nM	-	0.97 ± 0.44 (22.18)*	-	0.19 ± 0.08 (6.01)*	1.23 ± 0.39 (38.85)*	1.95 ± 0.88 (61.87)*
+Ko143 3 μM	0.05 ± 0.01 (1.06)	0.09 ± 0.05 (2.08)	0.03 ± 0.01 (1.01)	0.04 ± 0.07 (1.32)	0.04 ± 0.04 (1.32)	0.05 ± 0.18 (1.72)
SN-38	0.09 ± 0.01 (1.00)	0.61 ± 0.04 (6.72)*	0.02 ± 0.01 (1.00)	0.43 ± 0.21 (20.08)*	0.12 ± 0.05 (5.74)*	0.71 ± 0.86 (32.80)*
+OTS964 5 nM	-	0.67 ± 0.15 (7.36)*	-	0.49 ± 0.26 (22.94)*	0.11 ± 0.01 (5.14)*	0.81 ± 0.42 (37.73)*
+OTS964 10 nM	-	0.69 ± 0.18 (7.59)*	-	0.53 ± 0.09 (24.38)*	0.25 ± 0.12 (11.76)*	1.19 ± 0.74 (55.25)*
+Ko143 3 μM	0.09 ± 0.01 (0.96)	0.14 ± 0.05 (1.53)	0.03 ± 0.01 (1.24)	0.05 ± 0.47 (2.36)	0.03 ± 0.48 (1.21)	0.05 ± 0.03 (2.47)
Cisplatin	1.08 ± 0.23 (1.00)	1.22 ± 0.29 (1.12)	0.61 ± 0.12 (1.00)	0.81 ± 0.21 (1.34)	0.47 ± 0.40 (0.77)	0.93 ± 0.03 (1.53)
+OTS964 5 nM	-	1.22 ± 0.15 (1.13)	-	0.73 ± 0.04 (1.21)	0.40 ± 0.20 (0.65)	1.00 ± 0.13 (1.64)
+OTS964 10 nM	-	1.39 ± 0.13 (1.28)	-	0.82 ± 0.21 (1.34)	0.43 ± 0.04 (0.70)	0.80 ± 0.07 (1.31)
+Ko143 3 μM	1.11 ± 0.02 (1.02)	1.48 ± 0.06 (1.37)	0.71 ± 0.18 (1.17)	0.79 ± 0.36 (1.30)	0.48 ± 0.14 (0.79)	0.72 ± 0.26 (1.18)

* Indicated that the IC_50_ values of chemotherapeutic drugs in drug-resistant cell line had significant statistical difference from the counterparts in corresponding sensitive cell line without inhibitor (p < 0.05).

^a^ IC_50_ values were determined by modified MTT colorimetric assay, and are shown as mean ± SD.

^b^ Resistance fold (RF) were calculated by the IC_50_ values for chemotherapeutic drugs of drug-sensitive cells without inhibitor, divided by the IC_50_ values for chemotherapeutic drugs of drug-sensitive cells with inhibitor or drug-resistant cells in the absence or presence of inhibitor.

### OTS964 Upregulated ABCG2 Protein Expression Level.

**FIGURE 2:**
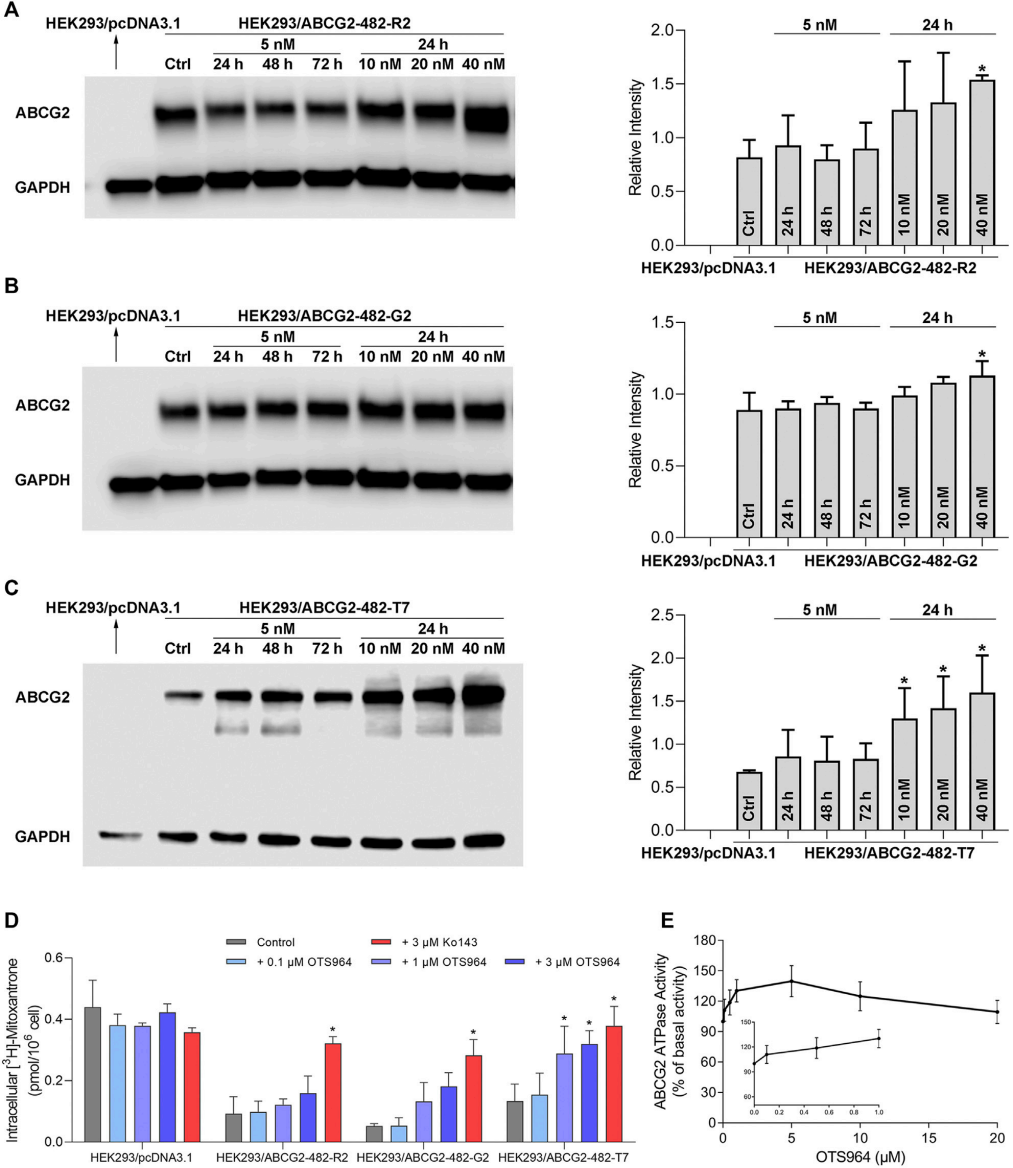
OTS964 enhances ABCG2 protein expression in gene-transfected HEK293 cells expressing ABCG2. The protein expression of ABCG2 in **(A)** HEK293/ABCG2-482-R2, **(B)** HEK293/ABCG2-482-G2, **(C)** HEK293/ABCG2-482-T7 cells after treatment with 5 nM OTS964 up to 72 h or incubation for 24 h with OTS964 at 10 nM, 20 nM or 40 nM concentrations. OTS964 inhibits transport function of ABCG2 in gene-transfected HEK293 cell lines. **(D)** The tritium-labeled mitoxantrone accumulation in ABCG2-transfected HEK293 cells (HEK293/ABCG2-482-R2, HEK293/ABCG2-482-G2, and HEK293/ABCG2-482-T7) and their corresponding drug-sensitive cells HEK293/pcDNA3.1. Ko143 functioned as a known inhibitor for ABCG2. OTS964 concentration-dependently stimulates ATPase activity of ABCG2 within 20 μM. **(E)** The effect of OTS964 on ATPase activity of ABCG2 in insect cell membrane vesicles was determined as described in materials and methods section. All data are exhibited as mean ± SD. *p < 0.05 compared with control group.

It is possible that upregulation of MDR-associated ABC transporters is responsible for increased resistance and reduced efficacy. Hence, a Western blot analysis was conducted to determine the protein expression level. As shown in Figures 2A–C, no significant change in ABCG2 expression was observed in HEK293 cells transfected with wild-type or mutant (R482G and R482T) ABCG2, followed by treatment with OTS964 at 5 nM up to 72 h. However, OTS964 at 40 nM had the ability to increase ABCG2 expression level in cells expressing wide-type and R482G-mutant ABCG2 (Figures 2A,B). According to Figure 2C, OTS964 concentration-dependently enhanced R482T-mutant ABCG2 protein expression followed by 24 h treatment in cells expressing R482T-mutant ABCG2.

### OTS964 Inhibited Transport Function of ABCG2.

To further elucidate the possible interaction between ABC transporter and OTS964, transport function mediated by ABCG2 was analyzed by an accumulation assay. OTS964 at 3 μM significantly increased intracellular accumulation level of [^3^H]-mitoxantrone from 29.6% to 72.6% in HEK293/ABCG2-482-T7 cell line (Figure 2D); however, there was no significant difference in [^3^H]-mitoxantrone accumulation in HEK293/ABCG2-482-R2 and HEK293/ABCG2-482-G2 cell lines relative to HEK293 cells transfected with an empty vector. Together, OTS964 at 3 μM could inhibit ABCG2-mediated efflux of mitoxantrone, which is an established substrate of ABCG2, in R482T-mutant-ABCG2-overexpressing cell line.

### OTS964 Stimulated ABCG2 ATPase.

To further evaluate interaction between OTS964 and MDR-associated ABC transporter, ABCG2-mediated ATP hydrolysis was measured in total membranes after incubation with serial concentrations of OTS964. OTS964 reached a maximum of 139.8% of basal activity for ABCG2 at 20 μM, and achieved 50% maximum stimulatory activity at 0.14 μM (Figure 2E). Hence, these results showed that OTS964 had a concentration-dependent stimulation of ATPase activity of ABCG2 transporter.

### Docking simulation of OTS964 in drug-binding pocket of human ABCG2.

**FIGURE 3:**
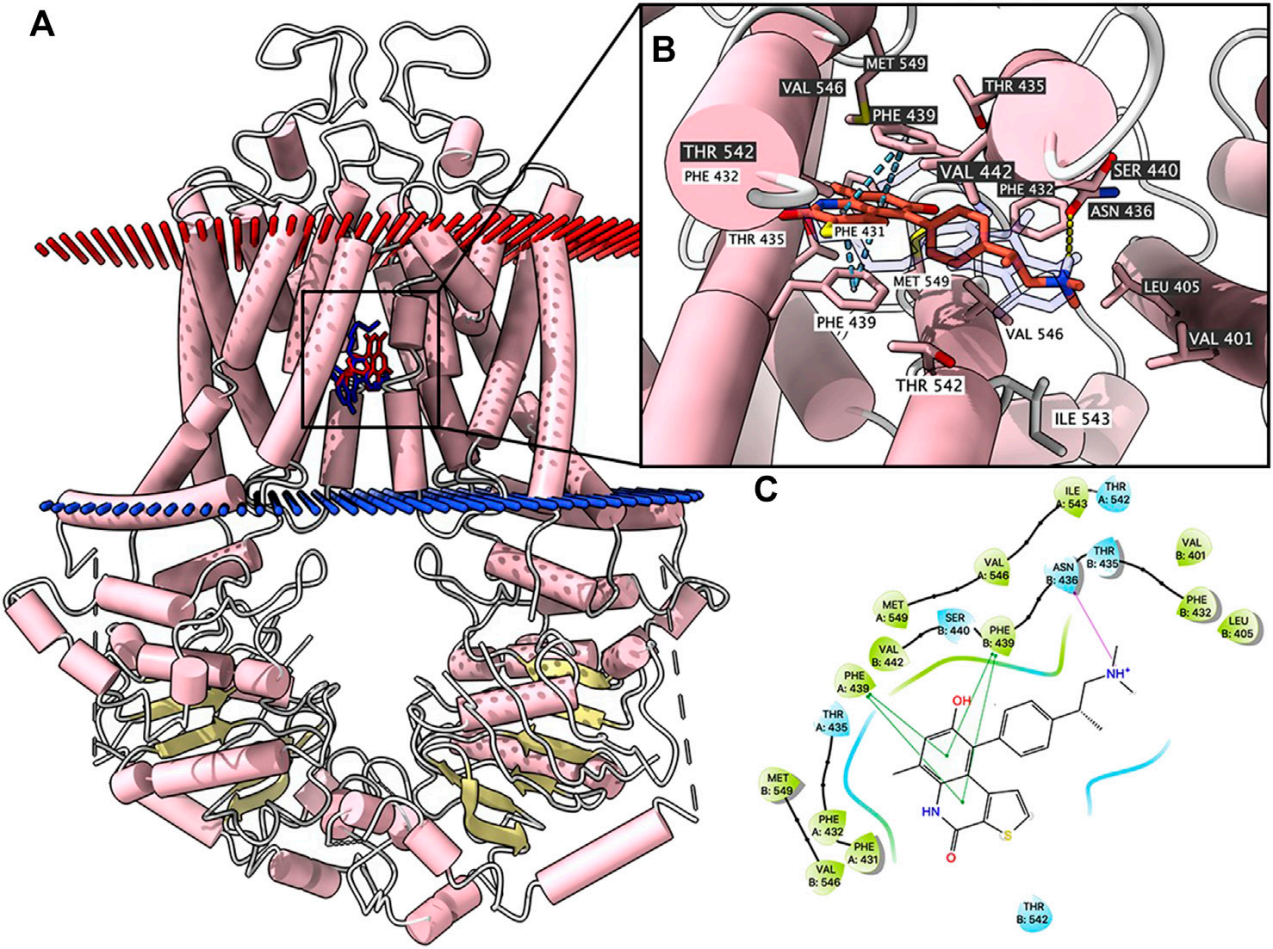
Highest scoring docked pose of OTS964 within human ABCG2 at substrate-binding site. **(A)** Overview of mitoxantrone and best-scoring pose of OTS964 in drug-binding pocket of ABCG2 protein. Mitoxantrone and OTS964 are displayed as colored sticks, blue: mitoxantrone; red: OTS964. **(B)** Details of interactions between OTS964 and ABCG2 binding pocket. Predicted bonds are displayed as colored dash lines: hydrogen bond: yellow; *pi-pi* stacking: blue. Labels with white or black background indicate chain A or B, respectively. **(C)** 2D OTS964-ABCG2 interaction. Important amino acids are displayed as colored bubbles (green: hydrophobic; blue: polar). Predicted bonds are displayed as colored lines: green line: *pi-pi* stacking; purple line with arrow: hydrogen bond.

According to ATPase results, OTS964 had a stimulatory effect due to its interaction at the drug-binding pocket. To assess this, we applied a docking simulation in the mitoxantrone-binding site of ABCG2 protein (6VXI). The results showed that OTS964 docked into ABCG2 substrate-binding site with an affinity score of -8.4 kcal/mol. Details of the ligand-receptor interaction are displayed in Figure 3. OTS964 is positioned and stabilized in a hydrophobic cavity formed by Phe431, Phe432, Phe439 (chain A), and Val442, Phe439, Phe432, Phe405 (chain B). Additionally, OTS964 was stabilized by *pi-pi* stacking interactions formed with Phe439 in both chains. The ionized amine group of OTS964 was stabilized by a hydrogen bond formed with Asn436. Similarly, the poses of docked OTS964 and mitoxantrone (docking score: −9.2 kcal/mol) overlapped, indicating that OTS964 possibly shares a similar binding site with ABCG2 substrates.

## Discussion

TOPK has emerged recently as a potential cancer-specific biomarker and a druggable therapeutic target (Herbert et al., 2018). Importantly, TOPK is highly expressed in multiple cancer types, while it is rarely detected in normal tissues except for some fetal tissues and germs cells; hence, TOPK inhibitors may impart minimal damage to normal tissues (Herbert et al., 2018; Hu et al., 2019). OTS964 is a highly potent TOPK inhibitor with tumor suppressive activity in lung cancer (Park et al., 2017) and ovarian cancer (Ikeda et al., 2016), and also serves as a template for synthesizing novel TOPK inhibitors (Hu et al., 2019). Hematologic toxicity, such as anemia and leukocytopenia, has been observed during OTS964 administration; this can be resolved by using a liposomal formulation (Matsuo et al., 2014; Gilabert-Oriol et al., 2019), which provides us with a clue that OTS964 may have off-target effects on indirect targets. In addition to having similar volumes of distribution as ABCG2, we postulated that the expression of ABCG2 might also influence the effectiveness of OTS964.

Our experiments with cell viability assay in drug-selected and gene-transfected cell lines indicated that OTS964 had high potency with IC_50_ values at the nanomolar level, which is consistent with previous researches (Matsuo et al., 2014; Pirovano et al., 2017). Furthermore, ABCG2 overexpression could confer resistance to OTS964 in cancer cells. As NCI-H460 is a lung cancer cell line, and S1 is a colon cancer cell line, it might, to some extent, develop other mechanisms of drug resistance apart from overexpressing ABCG2. Therefore, cells transfected with ABCG2 were used, in which ABCG2 was a solo contributor to MDR. Importantly, OTS964 resistance was observed in ABCG2-transfected HEK293 cell lines. It is known that switching arginine to glycine (R > G) or threonine (R > T) at amino-acid 482 in *ABCG2* gene may occur due to drug-induced mutation or genetic polymorphisms, which could cause substrate specificity and different resistance levels to substrate-drugs (Robey et al., 2003; Alqawi et al., 2004; Ejendal et al., 2006). Mitoxantrone is found to be a substrate drug of all ABCG2 variations; however, rhodamine 123, doxorubicin, and daunorubicin are transported by only mutant R482T or R482G but not by the wild-type R482 ABCG2 (Honjo et al., 2001; Robey et al., 2003). Also, compared with wild-type ABCG2, an R482G mutation confers relatively less resistance to SN-38 and topotecan (Honjo et al., 2001). Our results showed that ABCG2 variation at position 482 had some effect on OTS964 resistance. The RF values were higher in cells expressing R482G- and R482T-mutant ABCG2 relative to wild-type ABCG2. Thus, mutant ABCG2 (R482G or R482T) may confer higher level of resistance to OTS964 among all ABCG2 proteins. Together, we hypothesized that the efficacy of OTS964 could be compromised at the presence of ABCG2. Also, OTS964 induced resistance could be sensitized by an ABCG2 reference inhibitor, suggesting that ABCG2-overexpression is the mechanism of OTS964 resistance.

It is documented that some substrates of ABC transporters (Dai et al., 2008; Eadie et al., 2014), such as lapatinib, imatinib, nilotinib, and dasatinib, can compete with another drug substrate for transport function (Wu et al., 2020); as a result, a repurposed drug substrate has the ability to sensitize MDR-associated ABC transporters to another drug substrate. Considering the cytotoxic nature of OTS964, high concentrations (1 or 3 μM) used in accumulation assay would be too toxic and not usable clinically. Hence, antagonizing activity of OTS964 at non-toxic concentrations was evaluated only in MDR cell lines to avoid additive toxicity. In repositioning studies, our results indicated that rather than antagonizing MDR, OTS964 may enhance ABCG2-mediated MDR as evidenced by increasing IC_50_ values for many antineoplastic drugs after co-treatment with OTS964 at non-toxic concentrations (5 or 10 nM). We postulated that the absence of antagonizing effect on substrate-drug sensitivity might be the result of highly potent nature of OTS964 and high resistance level conferred by ABCG2. Of note, co-treatment with OTS964 did not affect drug sensitivity of cisplatin, which is a non-substrate drug for ABCG2, indicating that failure of sensitizing MDR cell lines to substrate-drugs may be specific to ABCG2. However, these findings do not warrant further testing of OTS964 as a reversal agent.

As different responses to OTS964 were found among ABCG2 variations above, gene-transfected HEK293 cell lines were used for mechanism-based studies. In some cases, upregulating the protein expression of ABCG2 promotes resistance and attenuates efficacy (Wu et al., 2020). Therefore, an immunoblotting analysis was performed to evaluate protein expression after OTS964 treatment. Attempts to circumvent the overlapping effect from additive cytotoxicity and to reduce the extent of off-target activity, cells were treated with a low concentration for a long time or with high concentrations for a short time, separately. At low concentration (5 nM), assuming negligible cytotoxicity, cell viability was more than 80% in MDR cell lines; by contrast, the highest concentration (40 nM) approached IC_50_ value in ABCG2-transfected cell lines. As a result, ABCG2 expression was not significantly changed after treatment with OTS964 at 5 nM up to 72 h. However, after 24 h incubation with OTS964 at 40 nM, higher expression level of ABCG2 was observed in MDR cell lines expressing ABCG2. Together, these may, at least in part, be the reason for the limited effectiveness of OTS964 and enhanced ABCG2-mediated MDR.

Tritium-labeled substrate accumulation was performed to investigate the effect of OTS964 on transport function conferred by ABCG2. Our results showed that OTS964 at high concentration (3 μM) could increase substrate-drug accumulation in MDR cell line (R482T-mutant-ABCG2 overexpression cells), but not in their corresponding parental cell lines. This effect might result from a high concentration of OTS964 competitively inhibiting the efflux of mitoxantrone while impeding the transport function of ABCG2, and in turn increasing intracellular substrate accumulation. The accumulation study was carried out with a short-term treatment (4 h), which protected cells from influence of cell viability and other cellular functions, although concentrations of OTS964 used for these experiments were higher than IC_50_ value.

ATP hydrolysis is an energy requirement for transport of substrate-drugs against concentration gradient by ABCG2 (Glavinas et al., 2008). Hence, an ATPase assay was conducted to assess effect of OTS964 on ABCG2 ATPase activity. Our results demonstrated that OTS964 concentration-dependently stimulates the ATPase activity of ABCG2. Subsequently, the *in silico* molecular docking was conducted to explore the interaction of OTS964 with ABCG2. The molecular docking was performed using atomic structures of mitoxantrone-bound ABCG2 (pdb.6VXI). The docking results showed that OTS964 shares similar binding sites with known substrates for ABCG2. Molecular docking combined with ATPase data suggested that OTS964 interacts with drug-binding pocket of ABCG2 and behaves as a substrate for ABCG2 transporter.

## Conclusion

OTS964 is susceptible to ABCG2-mediated drug resistance, and this effect can be antagonized by known ABCG2 inhibitor. ABCG2-conferred resistance to OTS964 can be explained by its stimulatory effect on ATPase activity and upregulated protein expression of ABCG2. Additionally, OTS964 at a high concentration inhibits transport function mediated by ABCG2; however, OTS964 at a low concentration promotes ABCG2-mediated MDR rather than antagonizing drug resistance. These findings may serve as a valuable foundation for follow-up clinical investigation on potential use of OTS964.

## Data Availability

The original contributions presented in the study are included in the article/Supplementary Material, further inquiries can be directed to the corresponding authors.
